# Updating the Chieti Affective Action Videos database with older adults

**DOI:** 10.1038/s41597-021-01053-z

**Published:** 2021-10-20

**Authors:** Pasquale La Malva, Irene Ceccato, Adolfo Di Crosta, Anna Marin, Mirco Fasolo, Riccardo Palumbo, Nicola Mammarella, Rocco Palumbo, Alberto Di Domenico

**Affiliations:** 1 Department of Psychological, Health and Territorial Sciences (DiSPUTer), University G. d’Annunzio - Via dei Vestini, 31 - 66100 Chieti, Italy; 2 Department of Neuroscience, Imaging and Clinical Science, University G. d’Annunzio - Via dei Vestini, 31 - 66100 Chieti, Italy; 3 Behavioral Economics and Neuroeconomics, Center of Advanced Studies and Technology (CAST), G. d’Annunzio University of Chieti-Pescara, Chieti, 66100 Italy; 4 Department of Neurology, Boston University, 150 South Huntington Avenue, Boston, MA 02130 USA

**Keywords:** Human behaviour, Human behaviour

## Abstract

Validation of the Chieti Affective Action Videos (CAAV) database was replicated with a sample of older adults (age range 65–93). When designing experimental studies of emotions, it is crucial to take into consideration the differences in emotional processing between young and older adults. Therefore, the main goal of the present study was to provide an appropriate dataset for the use of CAAV in aging research. For this reason, the CAAV administration and the data collection methodology was faithfully replicated in a sample of 302 older adults. All the 360 standardized stimuli were evaluated on the emotional dimensions of valence and arousal. The CAAV validation in an older adults’ population increases the potential use of this innovative tool. The present validation supports the use of the CAAV database in future experimental studies on cognitive functions in healthy and pathological aging.

## Abstract (summary)

**Table:** 

Measurement(s)	emotion - valence & arousal
Technology Type(s)	Self-Assessment Manikin scale (SAM)
Factor Type(s)	emotional video stimuli • age group
Sample Characteristic - Organism	Homo sapiens

Machine-accessible metadata file describing the reported data: 10.6084/m9.figshare.14992173

## Background & Summary

The Chieti Affective Action Videos (CAAV)^1^ is an innovative database of movie clips developed specifically for experimental research in psychology. The CAAV comprises a large number of emotional action videos rated for valence and arousal. The action videos are homogeneous in terms of length (15 seconds), brightness, and camera angle. All the videos include everyday life actions. Crucially, the innovative aspect of this tool consists in controlling two factors: a) the gender of the actors performing the action; and b) the point of view (POV) through which the action is carried out. In particular, an actor and an actress performed the same 90 actions both in first-person POV and in third-person POV, for a total of 360 emotional action videos. For each stimulus, the CAAV provides an emotional rating based on arousal and valence scores. Indeed, the database validation is based on the Dimensional Model of Emotions^2,3^ and specifically on the the circumplex model of affect^4^. Briefly, this model postulates that emotions can be identified based on their location along two dimensions: valence and arousal. The dimension of valence (i.e., pleasantness) differentiates positive (pleasant) from negative (unpleasant) emotional states. The dimension of arousal (i.e., activation) differentiates highly exciting and arousing states from calm and relaxing states. The rating of the CAAV database is based on this dimensional approach and provides a continuous and balanced distribution of the stimuli across valence and arousal. As a result, the CAAV allows the identification of action videos with intermediate scores on both dimensions, which can be classified as emotionally neutral. The CAAV has innovative features when compared to previous emotional databases^5–7^. Several databases explored the emotional dimensions with static and dynamic stimuli (e.g., words^8^, pictures^9^, sounds^10^, faces^11^ and movie clips^12^). Among those using dynamic emotional stimuli, many of them consists of a collection of stimuli extracted from movie scenes, which reduces the possibility to standardize (or experimentally control) specific features (e.g., duration, brightness, and camera angle). On the contrary, the CAAV’s movie clips were tailored to maintain constant these features. In addition, each action of the CAAV was performed by a female and a male actor in both first and third person POVs. Therefore, the CAAV provides a gender and POV balanced material. These features make the CAAV expressly well-suited to analyze the role of POV on individuals’ emotional response^13^. Furthermore, the CAAV may be especially useful to explore and avoid evaluation bias due to the actor’s gender^14,15^. These characteristics make the CAAV a highly ecological and immersive tool.

In the previous validation of the CAAV, we included only young adults. Therefore, we neglected age-related differences in valence and arousal ratings of the emotional stimuli^16,17^. Indeed, studies showed that older adults differ from younger adults in several aspects of emotional processing. For instance, older adults demonstrated greater stability of mood^18^, greater capacity for emotional regulation^19–21^, reduced autonomous reactions to emotional stimuli^22–26^, and reduced emotion recognition^27,28^. These differences could affect the CAAV ratings on valence and arousal. Some emotional action videos may be perceived as more or less positive or exciting by a sample of older adults. Supporting this possibility, previous studies found differences between young and older adults in the ratings of emotional images from the International Affective Picture System (IAPS)^9^ database^29,30^. For this reason, it is crucial that studies on aging utilize emotional stimuli rated by a sample of older people. Given the growing research interest on the role of emotions on cognitive functions in aging, the goal of this work was to replicate the CAAV validation with a sample of older adults. As a result, the appropriate valence and arousal scores will be provided for the use of an innovative, ecological, and immersive tool for experimental psychological research in aging.

## Methods

### Participants

The sample included 302 healthy older adults (65–93 years old, mean = 72.67, SD = 6.61) who participated on a voluntarily basis, receiving no compensation. All participants were Italian and Caucasian. They were all Italian native speakers and able to write and read. Specifically, we recruited 151 males and 151 females with years of education ranging from 0 to 24 (mean = 8.95 years; SD = 4.47). In order to detect the presence of cognitive impairment in older adults, the Mini Mental State Examination (MMSE)^31^ test was administered. The test consists of 30 items referring to seven cognitive domains: orientation in time, orientation in space, words encoding, attention and calculation, recall, language, and constructive praxia. The total score ranged from 0 to 30 points. The raw score is corrected according to the age and years of education of the participant. A corrected score below 24 indicates possible impairment of cognitive abilities. All participants achieved a corrected MMSE score of at least 24 (mean = 26.73; SD = 1.51). Participants received and signed a written informed consent before starting the experiment. Ethical approval was obtained by the Institutional Review Board of Psychology (IRBP) of the Department of Psychological, Health, and Territorial Sciences of the G. d’Annunzio University of Chieti-Pescara.

### Stimuli

All the 360 video clips in the CAAV database were used. The video clips presented 90 actions, balanced both by the perspective (third-person and first-person POVs), and the actor’s gender (male and female). The POV was manipulated to control for the immersivity of the emotional actions. In fact, stimuli in the first-person POV have been found to be more immersive and to elicit higher valence and arousal scores^32–34^. Notably, perspective taking ability changes across the lifespan, and age-related differences has been consistently found in previous studies^35,36^. Moreover, the actors’ gender was manipulated to control for potential gender-biases in the evaluations^37–39^. Therefore, the CAAV database comprises the same 90 actions performed by I) a male actor in the first person POV, II) a male actor in the third person POV, III) a female actor in the first person POV, and IV) a female actor in the third person POV. Both actors were 24 years old and worn a black shirt and a pair of blue jeans in each video.

Finally, different aspects were controlled when developing the CAAV such as: the presence of a few simultaneous elements in the scene, the variation of the camera angle, the light exposure, the setting, and the background.

Regardless the action that was performed, the length of the videos was kept constant (15 seconds). The movie clips do not contain any sound. Finally, each video shows a single straightforward action, so that all the stimuli are easy to encode even for older adult participants. We used the video stimuli in their original format (.mpg extension) and with a 1920 × 1080 resolution. For each video, we kept the same identification code originally assigned in the database. For more details on the stimuli creation, refer to the previous validation study^1^. An overview of the CAAV stimuli and the entire database are freely available for download on the Figshare platform^40^.

### Rating procedure

The rating procedure was the same used for the previous validation with the younger adults sample^1^. In fact, the goal of the present study was to carefully replicate the CAAV validation in an older adults sample. For this reason, the 360 action videos have been divided into four different lists (A, B, C, D). Each list contained 90 randomized actions. The lists were the same used in the previous validation study. The videos selected for each list were balanced by gender of the actor and POV. The four lists contained the same actions, but each list had one video of each action in one of the following four versions: (1) first person POV - male actor; (2) first person POV - female actor; (3) third person POV - male actor; (4) third person POV - female actor. Therefore, the same action was never repeated within each list. Based on the two dependent variables considered (valence and arousal), the total sample was divided into two groups (Table 1): one group rated the videos for valence, the other group rated videos for arousal. The four different lists were balanced among the participants of each group. The group that rated videos’ valence was composed by 141 participants (69 M/72 F), aged between 65 and 89 (mean = 72.47 years; SD = 6.13), with years of education between 0 and 18 (mean = 8.82 years; SD = 4.08), and an average corrected MMSE score of 26.73 (SD = 1.54). The group that rated videos’ arousal was composed by 161 participants (82 M/79 F), aged between 65 and 93 years (mean = 72.84 years; SD = 7.01), with years of education between 3 and 24 (mean = 9.07 years; SD = 4.79), and an average corrected MMSE score of 26.73 (SD = 1.49).

**Table 1:** Characteristics of the dataset.

Subjects	Experiment	Protocol	Data
141	Rating CAAV videos	Measurement of Valence dimension	Online-only Table 1
161	Rating CAAV videos	Measurement of Arousal dimension	Online-only Table 1

Study Characteristics.

### Rating tool

The tool we used for the evaluation of the movie clips was the Self-Assessment Manikin (SAM)^41^. The SAM is a non-verbal pictorial assessment technique commonly used in the study of emotions. Furthermore, the SAM technique is a simple and rapid administration tool that can be used efficiently in the older adult population^42^. This tool measures a person’s affective reaction to a stimulus based on the Dimensional Model of Emotions, namely the Circumplex Model. According to the Circumplex Model, the emotions are distributed in a two-dimensional circular space, containing valence and arousal dimensions^4^. Valence represents the horizontal axis and expresses the level of pleasure that ranges from negative to positive (left-right on the x-axis). Arousal represents the vertical axis and expresses the level of physiological activation from low to high (down-up on the y-axis). The SAM tool measures both these dimensions using two Likert scales. As in the previous CAAV validation, we used the 9-point scale version. Therefore, one group used the SAM to rate videos’ valence, where the value 1 corresponded to negative, 5 to neutral, and 9 to positive valence. The other group similarly used SAM to rate videos’ arousal, where the value 1 corresponded to low, 5 to medium, and 9 to high activation. We would like to highlight that by using the same rating tool adopted in the previous CAAV validation it is possible to directly compare older adults’ scores with their younger counterpart.

## Data Records

All the data obtained for the validation of the CAAV in the older adult sample can be downloaded on the Figshare platform^43^. In particular, the data are reported in an Excel file named “AgingCAAV_Dataset”. The data have been arranged in the same way as in the previous CAAV dataset to facilitate consultation and comparison between the two datasets^40^. The file contains the mean scores and standard deviations for both valence and arousal of all the 360 videos. These scores are available both for the whole sample and separated by gender of the participant. Furthermore, a file containing the raw data of all the experimental subjects is available in an additional Excel file named “AgingCAAV_RawData”. In the current dataset the MMSE scores (raw and corrected) and years of education have been added. Consequently, the file contains the following variables: subject ID, gender, age, education (in years), MMSE_Raw, MMSE_Correct, list administered, and the valence/arousal rating for each of the 360 videos.

## Technical Validation

**Fig. 1:**
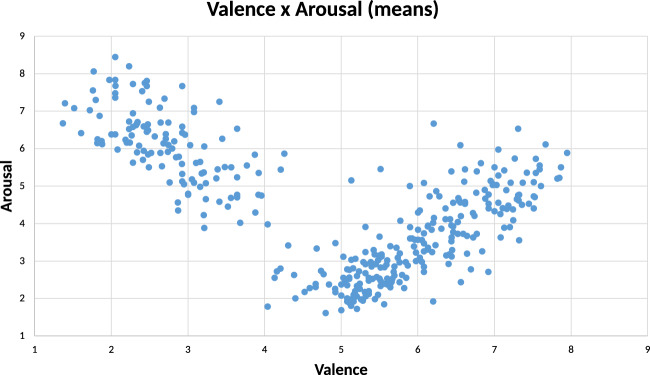
Scatterplot of the interaction between valence and arousal scores of each video. The average valence score is reported on the X axis, while the average arousal score is reported on the Y axis for each video.

Regarding the methodological reliability, both the administration procedure and the instruments used were the same as the previous CAAV validation^1^. The rating was performed using a laptop. In particular, the video stimuli were presented using the E Prime 2.0 software, which allowed to randomize the presentation of the 90 stimuli within each list. Each participant carried out the rating task in a quiet room. Lighting conditions were kept constant among all participants. Before starting the rating task, a print version of the MMSE was administered to each participant. Subsequently, the participant was placed in front of the laptop screen where s/he performed the rating task. Before starting the task, three tutorial videos (“play with a balloon”, “waving a fan” and “punch a wall”) were presented, with both POVs (first/third POV) and actors (female/male). In this way, the participants were able to familiarize with the type of stimuli and the rating method. These videos are not included in the official database as they were used for demonstration purposes only. Once the tutorial session was completed, the participant was ready to start the main session of the task. Each video was preceded by the phrase “Please rate the next video” that stayed on the screen for three seconds. Immediately after, the movie clip was presented for its entire duration (15 seconds). When the video ended, it disappeared from the screen, and the image of the SAM tool appeared. Specifically, in the valence group the SAM instructions were “Please rate the video based on valence”, while for the arousal rating group the SAM instructions were “Please rate the video based on arousal”. Participants could express their ratings by pressing the corresponding key number (1–9). The interplay between the mean scores of valence and arousal is plotted in Fig. 1. A U-shaped distribution emerged along the valence and arousal dimensions continuum, with greater arousal for negative (i.e., low valence) and positive (i.e., high valence) actions, and lower arousal for neutrals (i.e., average valence) actions, similar to what was found in the previous CAAV validation. The entire administration procedure lasted about 45 minutes.

## Usage Notes

By validating the CAAV database with a sample of older adults, the emotional video stimuli can be selected and used more adequately for experimental studies on aging. Indeed, the literature shows that across the life span the way people process emotional information changes^44–48^. Consequently, the rating data collected in the older adult sample would allow the stimuli of the CAAV to be more suitable for aging research. The provided dataset encourages new experimental studies on emotions that will investigate differences between young and older adults. Furthermore, these original and innovative video stimuli could be useful for studies on cognitive functions in general (attention, perception, memory, etc.) and emotions in both healthy and pathological aging^49–51^. Emotional action videos of the CAAV could be also used for the development of both mood induction methodologies and emotional regulation training programs^52,53^. The availability of a well-matched and highly controlled database of video stimuli that explicitly manipulates different perspectives (third/first person POV) opens new avenues for ecological studies^54–56^. For instance, it would be interesting to use the CAAV stimuli through augmented reality instruments (e.g., with Virtual Reality tool). To further increase the immersion factor, the database could be developed further by adding new stimuli where the action videos are performed by older adult actors (both male and female). For example, the FACES database provides images of facial emotional expressions of young, middle, and older adults^57^. A shared age between the actors in the videos and the participants who observe the stimuli could modulate the assessment of the valence and arousal of the observed actions by further increasing the emotional involvement of older adult.
